# Allogenic adipose derived mesenchymal stem cells are effective than antibiotics in treating endometritis

**DOI:** 10.1038/s41598-023-36820-y

**Published:** 2023-07-12

**Authors:** Vinay Bhaskar, Sikander Saini, Shama Ansari, Shubham Ghai, Abhishek Thakur, Suman Chopra, Vivekananda Verma, Dhruba Malakar

**Affiliations:** Cell and Molecular Biology Lab, Animal Biotechnology Centre, National Dairy Research Institute, Karnal, India 132001

**Keywords:** Adult stem cells, Mesenchymal stem cells, Regeneration, Antimicrobial responses, Cytokines, Gene regulation in immune cells, Infection, Cell delivery, Regenerative medicine, Stem-cell biotechnology

## Abstract

Endometritis is a uterine inflammatory disease that causes reduced livestock fertility, milk production and lifespan leading to significant economic losses to the dairy industry. Mesenchymal stem cells (MSC) may act as an alternative for inefficacy of antibiotics and rising antibiotic resistance in endometritis. The present study aimed to cure the chronic endometritic buffaloes using allogenic adipose-derived MSCs (AD-MSC). AD-MSCs were isolated from buffalo adipose tissue and characterized by multilineage differentiation as well as MSC-specific markers. The in vivo safety and efficacy were assessed after infusion of AD-MSCs. In safety trial, cells were administered in healthy buffaloes via different routes (IV and IC) followed by examination of clinical and hematological parameters. In efficacy study, AD-MSCs treatments (IV and IC) and antibiotic therapy (ABT) in endometritic buffaloes were comparatively evaluated. AD-MSCs did not induced any immunological reaction in treated buffaloes. PMN count, CRP levels and VDS were significantly (*p* ≤ 0.05) reduced after AD-MSCs infusions in IV and IC groups and no significant difference was observed in antibiotic group. The IV group was marked with 50% absolute risk reduction in endometritis and 50% live calf births after artificial insemination in comparison with ABT group. Anti-inflammatory cytokines (*IL4* and *IL10*) and anti-microbial peptides (*PI3*, *CATHL4*, *LCN2* and *CST3*) expressions were significantly (*p* ≤ 0.05) upregulated in IV group. The calf delivery rate after the treatments in IV group was higher (50%, 3 calves) than the other groups (IC: 33.3%, 2 calves; ABT: 16.6%, 1 calf). In conclusion, the administration of AD-MSCs through IV route was found to be safe and efficacious for alleviating chronic endometritis in dairy buffaloes.

## Introduction

Endometritis is one of the most important postpartum disorders, defined as inflammation of the endometrium marked by postpartum viscous vaginal discharge that persist for more than 21 days. It is caused by a variety of bacteria viz. *Fusobacterium necrophorum*, *Fusobacterium nucleatum*, *Escherichia coli*, *Porphyromonas levii*, *Trueperella pyogenes* and *Bacteroides pyogenes*^1^. Failure to eliminate such infection may lead to reduced fertility, milk production and substantial dairy economic losses^1,2^. Endometritis has been shown to disrupt luteal development and function, which leads to suboptimal fertilization and increased pregnancy loss^3,4^. The infection due to endometritis triggered extended postpartum anestrous periods (6 months) in buffaloes, with persisting corpora lutea in 28% of instances and inactive ovaries that appeared to be devoid of large follicles and corpora lutea in 72% of cases^5^. In addition to reproduction, endometritis also has a negative impact on milk production^6,7^. A significant positive correlation between the prevalence of endometritis and reduced milk yield (15% reduction per day) has been demonstrated in zero-grazed dairy herds^8^. The resultant significantly longer calving intervals, low milk yields, and decreased reproductive efficiency drive up the culling rates in dairy herds^9^. Due to the inherent tendency of buffalo towards wallowing, prevalence rate of endometritis around the world ranges from 3 to 50%^10^. Given a share of 15% of total worldwide milk production by buffalo^11^, the net economic loss due to endometritis represents a significant concern. Nonetheless, the extensive use of antibiotics to treat metritis generally achieved sub-optimal healing^12–15^. In addition, using antibiotics often results in the development of anti-microbial resistance in animals^16–18^ and the persistence of antibiotic residues in milk, both of which pose a significant risk.

Mesenchymal stem cells (MSCs) are a type of adult stem cells that have the potential of indefinite self-renewal, differentiation into multiple cell lineages and immunomodulation. Conversely, MSCs does not impart adverse immune reaction^14^ and modulate the immune system through paracrine interaction with innate immunity cells along with secretion of soluble factors. Briefly, MSCs exhibit anti-inflammatory actions via inhibition of proliferation of dendritic cells, natural killer cells, T- and B-lymphocytes ^21–23^. A variety of secreted factors, such as transforming growth factor β, prostaglandin E2, interleukin-10 (IL10), indolamine 2,3 dioxygenase and tumor necrosis factor-stimulated gene-6, have been reported to aid the immunomodulatory activities of MSCs ^24^. Besides the indirect modulations, MSCs actively mitigate the pathogen load by secreting multiple antimicrobial proteins like LL-37 β-defensin, lipocalin-2 (LCN2) , cystatin C (CST3), IDR-1, elafin (peptidase inhibitor, PI3), hepcidin and cathelicidin-4 (CATHL4)^25–27^. The aforementioned reports along with others^28–30^ advocates the effectivity of MSCs against infections.

In recent years, stem cell therapy employing allogenic MSCs has emerged as an alternative approach to counter a variety of diseases in livestock^31–35^. The serious negative impact of endometritis on the productivity of dairy buffalo and the immunomodulatory potential of MSCs as well as their ability to inhibit polymicrobial infections prompted us to design this study. We hypothesized that administering AD-MSCs intravenously (IV) and intracervically (IC) to buffaloes with endometritis would enable the delivery of MSC-derived bioactive components at sites of infection, where they may immunomodulate the local immune responses, strengthen antimicrobial defenses against the disease pathogen, and eventually promote the native regenerative process of the damaged tissues. The focus of the present study was to isolate the MSCs from buffalo adipose tissue without enzymatic treatment, followed by its characterization according to the criteria laid down by the international society for cell and gene therapy (ISCT)^36^. Keeping in view the potential risks and benefits associated with stem cell therapy, we also investigated the safety and efficacy of these stem cells for chronic endometritis treatment in buffalo (*Bubalus bubalis*).

## Results

### AD-MSCs grow in monolayer and form colonies when cultured in vitro

**Figure 1:**
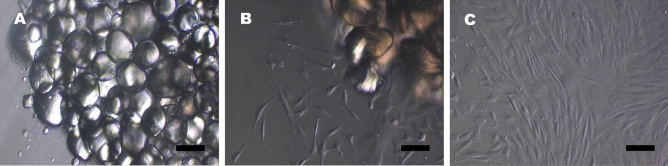
In vitro culture of AD-MSCs. (**A**) fat tissue explant on the culture dish**.** The fat globules are visible at this magnification. (**B**) AD-MSCs attaching to the surface of culture dish and growing in numbers. (**C**) AD-MSCs cultured in vitro and observed on day 20. Scale Bar represents 50 µm.

As a primary requirement of the study, AD-MSCs were isolated and in vitro cultured until 80% confluency. We isolated the cells using explant culture (Fig. 1A). Cells began to adhere as monolayer to the surface of culture flask and a steady growth with fusiform to polygonal fibroblast-like morphology was observed after at about 2 weeks of explant seeding (Fig. 1B). Cells were passaged after 80% confluency (Fig. 1C).

### AD-MSCs produce ALP and differentiate into multilineage cells

**Figure 2:**
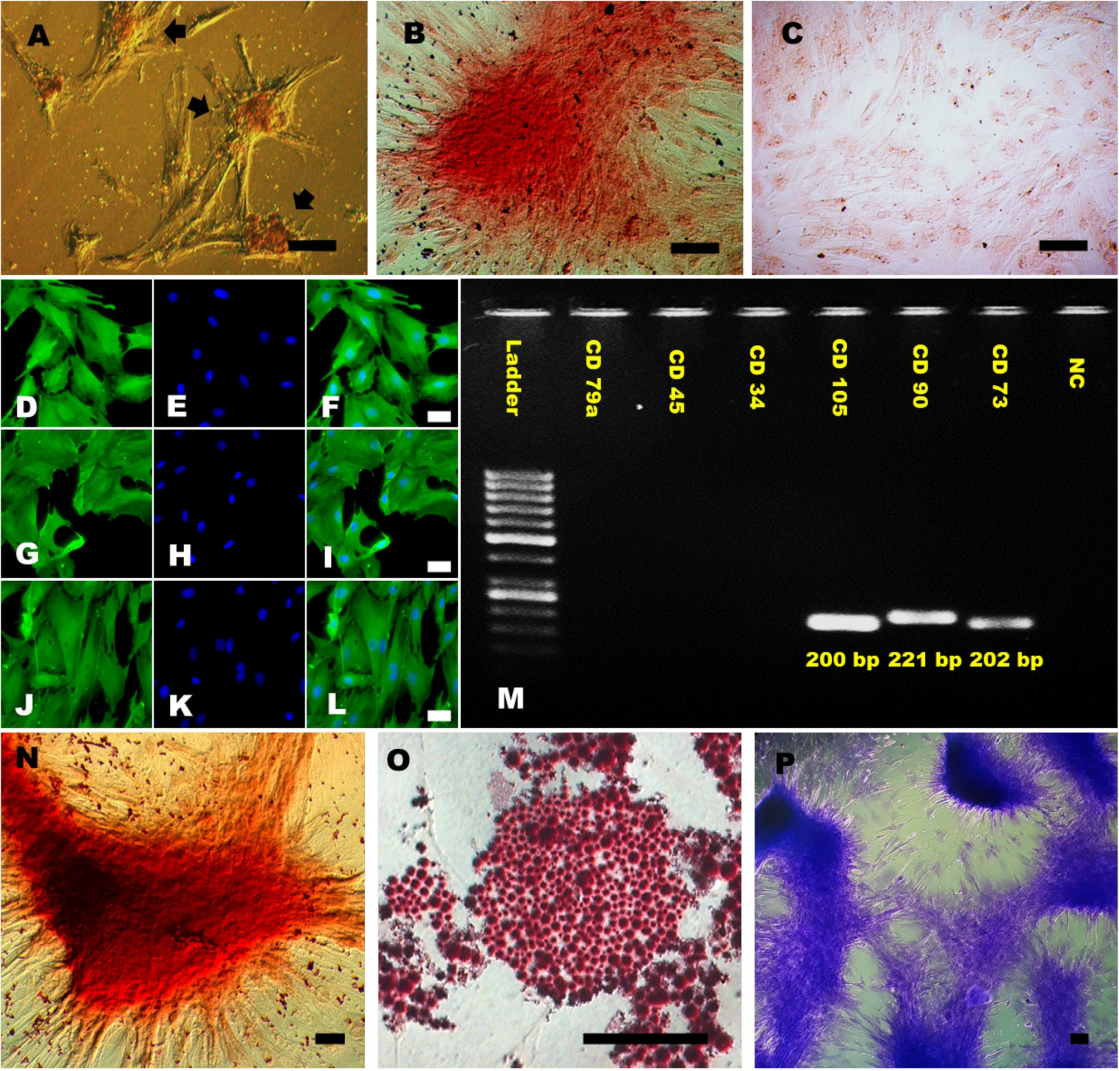
Characterization of AD-MSCs. (**A**) Colony Forming Units (CFU) as obtained after seeding low density of AD-MSCs, where the arrows represent distinctive colonies. (**B, C**) Alkaline phosphatase staining: Darkly stained MSCs (**B**) and Lightly stained fibroblast cells (**C**). (**D–L**) Immunostaining of AD-MSCs against characterization markers: anti-CD90 (**D–F**), anti-CD73 (**G–I**) and anti-CD105 (**J–L**). FITC filter (**D, G, J**); Nuclei stained with Hoechst 33342 dye (**E, H, K**); Merged (**F, I, L**). (**M**) RT-PCR based characterization of AD-MSCs. Expression of positive cell markers: *CD73*, *CD90* and *CD105* and expression of negative markers: *CD34*, *CD45* and *CD79a*. 100 bp ladder was used as ruler. *NC* Non-template control. (**N–P**) Multiple mesodermal lineage differentiation. Osteogenic lineage differentiated cells stained with Alizarin Red S dye characterized by prominent brick red color (**N**). Adipogenic lineage differentiated cells stained with Oil Red O dye characterized by red-colored fat droplets (**O**). Chondrogenic lineage differentiated cells stained with Toluidine Blue dye which imparted dark blue colour to AD-MSCs (**P**). Scale bar represents 50 µm.

For estimation of the proliferative and clonogenicity of MSCs in culture, AD-MSCs were seeded in low densities (i.e., 5 × 10^3^ cells/60 mm petri dish) and cultured. Eventually, AD-MSCs formed colonies with outward radiating cells at day 5–10 of the in vitro culture (Fig. 2A). In order to characterize, AD-MSCs were subjected to multiple mesodermal lineage differentiation at third passage. Differentiation into adipocytes, chondrocytes and osteocytes was achieved by culturing cells in different differentiation media containing specific biochemicals. When stained for alkaline phosphatase (ALP) activity, these colonies were observed to retain the dark red stain (Fig. 2B) as compared to the fibroblasts (Fig. 2C) which were lightly stained. The results indicated a fairly high ALP activity in AD-MSCs.

AD-MSCs cultured in the osteogenic differentiation medium a shift in the cellular morphology from a fibroblastic to cuboidal shape. The cells, during the culture period, started depositing calcium that were verified by a brick red color after alizarine red S staining (Fig. 2N). The control culture retained their fusiform morphology with inability to take up stain. When conditioned with adipogenic differentiation medium, AD-MSCs transformed into cell containing varying sizes of densely populated lipid vacuoles, which retained the oil red O stain (Fig. 2O). Whereas, the control culture showed few sparse cells forming vacuole and getting stained. After induction with chondrogenic differentiation medium, AD-MSCs transformed into a packed and rounded morphology. Toluidine blue staining which stains the glycosaminoglycans (GAG), imparted dark blue color to differentiated cells (Fig. 2P) and a very fade blue color in control cells. This signified a greater amount of accumulation of GAGs in AD-MSCs as compared to control cells with no induction. These findings show that the isolated cells are capable of ALP production and differentiating into various mesodermal lineages, which are key features of mesenchymal stem cells.

### AD-MSCs displayed positive mesenchymal stem cell markers

Based on the norms defined by the ISCT, a set of positive and negative markers were used to characterize the cells. AD-MSCs were evaluated by RT-PCR to be positive for CD73, CD90, CD105 and negative for CD34, CD45, CD 79a gene expression (Fig. 2M). The positive expression of markers was further confirmed by positive immunostaining with anti-CD73, anti-CD90, anti-CD105 antibodies (Fig. 2D–L).

### Intravenous and intracervical administration of allogenic AD-MSCs in healthy buffaloes induce no clinical effect or immune rejection

After the characterization, safety of the allogeneic AD-MSCs for the treatment of endometritis in buffalo was examined. Briefly, stem cells were introduced intravenously and intracervically in the healthy buffaloes on Day-0 and Day-7. Blood was collected from jugular vein for evaluation of clinical and hematological parameters at day-0, 1, 3, 7 and 15. Clinical parameters viz. RR (respiratory rate), HR (heart rate) and RT (rectal temperature) were within the reference range and remained stable (*p* > 0.05) after injection of AD-MSCs. The reference range for the same were adapted from elsewhere^37^. Hematological variables viz. hemoglobin, PCV (packed cell volume), leukocyte, erythrocyte, neutrophil, lymphocytes, monocytes and eosinophil levels were also within the reference range and demonstrated no significant difference (*p* > 0.05) between sampling days (Table 1). Additionally, the basophils and ESR (erythrocyte sedimentation rate) levels between the sampling days did not alter significantly (*p* > 0.05). In total, the administration of allogenic AD-MSCs into healthy animals did not alter the clinical or hematological parameters, indicating stable health with no signs of immune rejection of transplanted AD-MSCs.

**Table 1:** Clinical and hematological parameters in healthy dairy buffaloes treated with intravenous and intracervical suspension of AD-MSCs.

Parameters^a^	Reference^b^	Day0^c^	Day1	Day3	Day7^c^	Day15	*p* values
RR (bpm)	12–36	30.2 ± 0.2	29.8 ± 1.4	29.3 ± 1.3	29.5 ± 0.8	31 ± 1.5	0.2015
HR	40–80	54.2 ± 0.3	53.5 ± 1	53.6 ± 0.4	52.3 ± 0.7	54.5 ± 1.4	0.2541
RT (°C)	38–39	37.4 ± 0.5	37.7 ± 0.2	38.2 ± 0.3	38.4 ± 0.1	37.7 ± 0.2	0.2079
HEMOGLOBIN (g/dL)	8–15	11.2 ± 0.9	10.9 ± 0.1	11.1 ± 0.04	11 ± 0.1	11.2 ± 0.1	0.0599
PCV %	24–46	29.1 ± 0.1	29 ± 0.07	29.1 ± 0.1	29 ± 0.1	29.1 ± 0.1	0.0706
LEUKOCYTE (1 × 10^3^/µL)	4–12	10.6 ± 0.1	10.5 ± 0.07	10.6 ± 0.09	10.6 ± 0.07	10.6 ± 0.07	0.215
ERYTHOCYTE (1 × 10^6^/µL)	5–10	6.2 ± 0.05	6.07 ± 0.02	6.1 ± 0.02	6.1 ± 0.08	6.1 ± 0.06	0.9371
NEUTROPHIL (1 × 10^3^/µL)	0.6–4	3.1 ± 0.1	3 ± 0.09	3.1 ± 0.09	3.3 ± 0.15	3.5 ± 0.2	0.1381
LYMPHOCYTES (1 × 10^3^/µL)	2.5–7.5	5.4 ± 0.1	5.3 ± 0.09	5.4 ± 0.06	5.5 ± 0.1	5.6 ± 0.1	0.6576
MONOCYTES (1 × 10^3^/µL)	0.025–0.84	0.08 ± 0.03	0.1 ± 0.06	0.1 ± 0.04	0.1 ± 0.05	0.8 ± 0.03	0.2301
EOSINOPHIL (1 × 10^3^/µL)	0–2.4	1.3 ± 0.08	1.4 ± 0.06	1.45 ± 0.09	1.5 ± 0.05	1.2 ± 0.17	0.8021
BASOPHIL		0	0	0	0	0	0
ESR (mm/hr)		39.8 ± 1.2	42.1 ± 1.7	42.3 ± 1.7	41.6 ± 0.9	42.5 ± 1.1	0.3407

^a^*RR* respiratory rate, *HR* heart rate, *RT* rectal temperature.

^b^Reference values were adopted from Smith, 2020^37^.

^c^2 × 10^7^ cells were administered intravenously and intracervically on day-0 and day-7. Repeated measures one-way ANOVA (n = 6) with Tukey’s post hoc test was performed using GraphPad PRISM 8.

### Intravenous and intracervical administration of allogenic AD-MSCs in buffaloes with endometritis demonstrated significant reduction in inflammation

To determine the therapeutic efficacy, we administered allogenic AD-MSCs in buffaloes through different routes viz. jugular vein (IV) and intracervical (IC). Clinical parameters were evaluated at day 0, 1, 3, 7 and 15. The inflammatory marker, C-reactive protein (CRP) was quantified in blood serum collected at day-0 and -15. Additionally, cervical vaginal fluid was also collected at day 0 and Day 15 for vaginal cytology analysis by Giemsa staining and vaginal discharge score (VDS) analysis.

**Figure 3:**
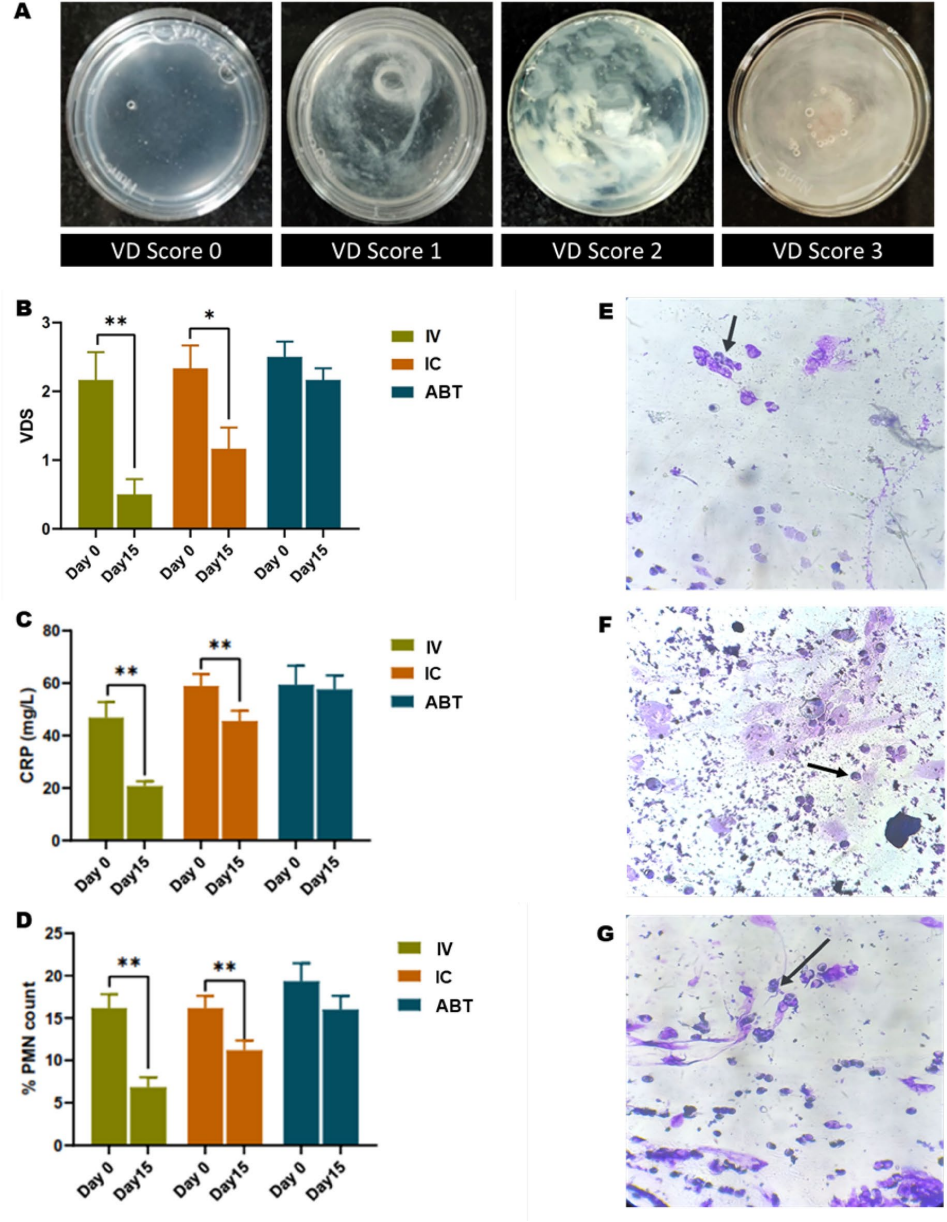
Effect of different treatments of AD-MSCs on Vaginal discharge score (VDS), C-reactive protein (CRP) levels and Polymorphonuclear leukocytes (PMN) count over 15 days period. (**A**) VDS scoring system. 0 = clear discharge, 1 = mucus with white flacks along with off-white pus, 2 = discharge containing < 50% pus and 3 = discharge containing > 50% pus. Higher score represents the high severity of infection in endometrium. (**B**) Mean VDS score of different interventions plotted against treatment period. (**C**) Mean CRP levels in serum of animals undergoing different treatments at day 0 vs. day 15. (**D**) Mean PMN count in vaginal fluid cytology of animals under different therapeutic treatments compared between day-0 and day-15. IV- Intravenous. Two-tailed paired t-test was performed with 6 animals in each group. *p ≤ 0.05; **p ≤ 0.01. (**E–G**) Vaginal fluid cytology for IV (**E**), IC (**F**), and ABT (**G**) groups, stained with Giemsa at day 15. Arrow indicates PMNs.

Clinical parameters (HR, RR, RT) in all the treatment groups remained stable (*p* > 0.05) between days after treatment (Table 2). However, the VDS values significantly dropped in IV and IC groups in contrast to the ABT groups at 15 days after treatment (Fig. 3B). Upon further analysis of VDS score data (Table S2), assuming antibiotic treatment group as baseline, ARR (absolute risk reduction) from metritis was calculated to be significant (50%) in IV group, as compared to IC (16.67%) group. In comparison to day-0, PMN count was also recorded significantly (*p* > 0.01) low at day-15 in both IV and IC groups (Fig. 3D–G). ABT treatment group did not demonstrate significant variation in PMN count. Moreover, CRP levels in the blood serum significantly (*p* > 0.01) reduced in IV and IC treatment groups (Fig. 3C) as compared to antibiotic-treatment (ABT) group at Day 15. The reduction in CRP levels and PMN count indicates a decreased infection in animals undergoing IV and IC treatment. Whereas, the overall healing of the endometrium inversely corresponds to the VDS score, which is significantly lowest in the IV group followed by IC group.

**Table 2:** Clinical parameters of endometritic buffaloes treated with AD-MSCs via different routes.

Parameters^a^	Treatment group	Day 0^b^	Day 1	Day 3	Day 7^b^	Day 15	*p* value
RR	IV	30.3 ± 0.8	31. ± 0.9	31 ± 0.9	30 ± 0.9	31 ± 0.9	0.9951
	IC	30.3 ± 0.8	30 ± 0.9	31 ± 0.9	29.8 ± 0.8	29.1 ± 1.2	0.6821
	ABT	30.8 ± 1.1	29.3 ± 1.4	30.1 ± 0.8	31 ± 1	30.5 ± 1.5	0.2965
HR	IV	53.5 ± 1.6	31.3 ± 1.3	54.8 ± 1.2	54.1 ± 0.8	51.1 ± 1.1	0.6864
	IC	52.8 ± 1.3	56 ± 1.9	55 ± 1.4	52.3 ± 1.5	51.6 ± 1.3	0.3453
	ABT	55.5 ± 1.1	53.6 ± 1	55.5 ± 1.2	52.8 ± 0.9	53.5 ± 0.5	0.3314
RT	IV	38.4 ± 03	54.1 ± 0.8	37.9 ± 0.12	38.4 ± 0.1	37.9 ± 0.2	0.7974
	IC	38.5 ± 0.3	55 ± 1.2	38.1 ± 0.3	38.6 ± 0.2	37.9 ± 0.1	0.8971
	ABT	39 ± 0.2	38.5 ± 0.2	38.8 ± 0.2	38.6 ± 0.2	37.9 ± 0.2	0.7946

^a^*RR* respiratory rate, *HR* heart rate, *RT* rectal temperature.

^b^2 × 10^7^ cells were administered on day-0 and day-7. IV = intravenous infusion, IC = intracervical infusion, ABT = antibiotic therapy. Values are means ± SD with six animals in each group. The repeated measures one-way ANOVA was performed and significant differences between the groups were evaluated by Tukey’s post hoc test.

### Intravenous administration of AD-MSCs causes significant increase in systemic expression of anti-inflammatory cytokines and anti-microbial peptides

**Figure 4:**
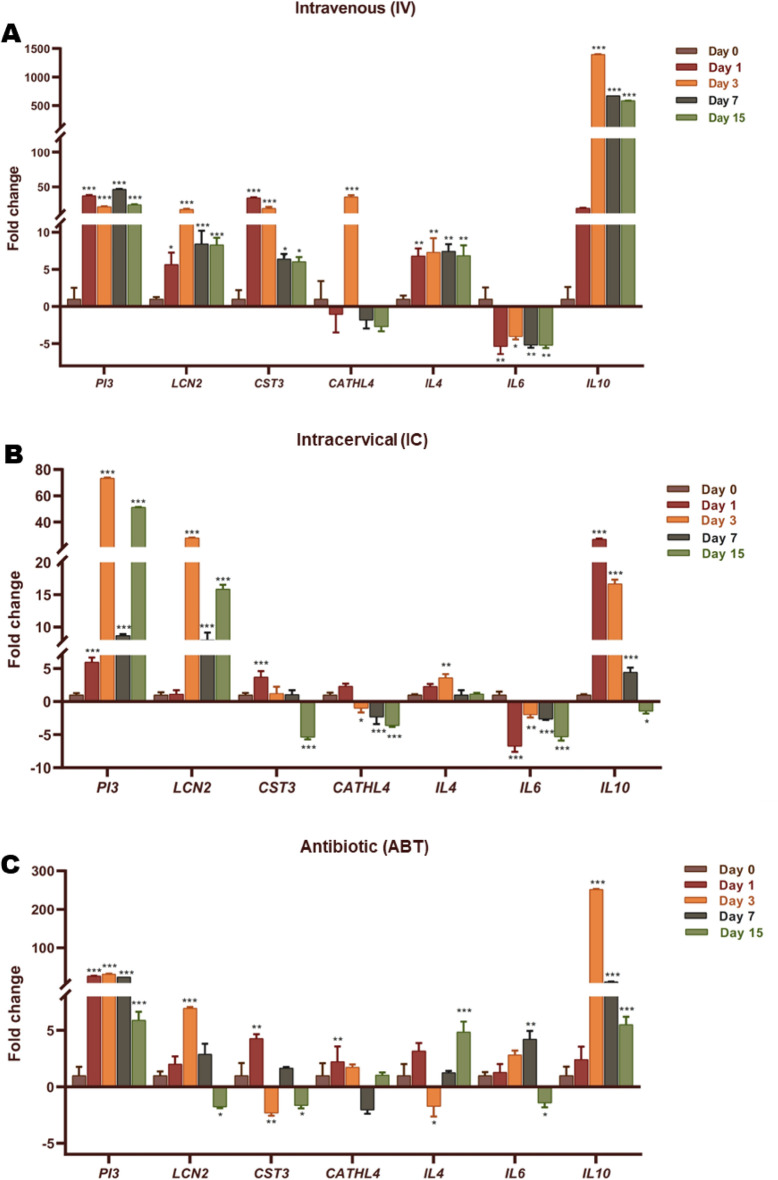
Gene-expression of anti-inflammatory, anti-microbial peptides and pro-inflammatory genes after different therapeutic interventions in buffaloes suffering from endometritis. The fold change in expression of anti-inflammatory (*IL4*, *IL10*), pro-inflammatory (*IL6*) and anti-microbial peptide (*PI3*, *LCN2*, *CST3*, *CATHL4*) genes in PBMCs of buffaloes suffering from endometritis. (**A**) The expression of genes in IV, (**B**) IC and, (**C**) ABT groups were analyzed for all the days with respect to Day 0 (control). The X-axis represents the various genes and Y-axis represents the fold change in gene expression. Bars represent mean number of transcripts (± SEM) of three repeats. Bars that have asterisk mark are significantly different (*p ≤ 0.05; **p ≤ 0.01; ***p ≤ 0.001) by one-way ANOVA with Dunnett post hoc test.

In order to investigate the effects of different modes of AD-MSCs administration on the expression of immunological and anti-microbial markers, we performed qPCR of *IL10*, *IL4*, *IL6*, *PI3*, *LCN2*, *CST3* and *CATHL4* genes on PBMCs isolated from IV, IC and ABT administered animals. Interleukins, IL10 and IL4 are the anti-inflammatory markers and IL6 is the pro-inflammatory marker. PI3, LCN2, CST3 and CATHL4 are the anti-microbial peptides released in response to microbial infection. The expression of all the genes was assessed relative to day 0 (Fig. 4).

The expression of *IL10* was significantly upregulated in all the groups after day 0 treatment. However, in IV group its expression on day 0 elevated up to 1500 folds on day 3 as compared to IC and ABT groups with 18 and 250 folds respectively. The expression of *IL4*, significantly increased after treatment at day 0 in IV group (7-folds) and remained approximately stable throughout the observation period (15 days). Whereas, its expression in IC group first increased significantly on day 3 which, thereafter, returned to basal level. The levels of *IL4* did not significantly increase until day 15 in ABT group. *IL6* expression did not vary significantly in IV and IC groups throughout the experimental period. Conversely, its expression increased gradually, reaching significant levels at day 7 and then decreased to non-significant value at day 15 in ABT group.

The expression levels of anti-microbial peptide *PI3* increased significantly in all the groups after day 0, with its levels reaching up to 75 folds in IC group. *LCN2* increased significantly in IV and IC groups and its expression remained elevated throughout the 15-day period. In ABT group, *LCN2* rose to significant levels followed by downregulation to below basal levels at the end of 15-day trial period. The expression of *CST3* initially increased significantly on day 1 in IC and ABT groups, which gradually downregulated to non-significant levels at day-15. However, its expression in IV groups significantly hiked up to 47 folds and maintained the significant levels till the end of study period. *CATHL4* expression did not varied significantly throughout the study in IC and ABT groups. Conversely, its expression in IV group significantly upregulated to 50 folds.

It is noteworthy, here, that the expression of *IL10* was much higher in the IV group as compared to the other treatments and its expression is maintained at around 500-fold during the treatment period. The expression of *IL4*, although around 7-folds, remained at significant levels throughout the trial period in IV group. *LCN2* remained significantly upregulated in only IC and IV treatment groups for the duration of 15 days. Briefly, *IL10*, *IL4*, *LCN2* and *CST3*, upregulated significantly during the treatment as compared to their scattered expression between days in other treatment groups.

### A higher proportion of delivered calves were obtained in buffaloes treated with intravenous administration of AD-MSCs

In order to evaluate the improvement in reproductive efficiencies, the buffaloes in different treatment groups were subjected to AI procedures after the end of 15 days experimental period. Calving percentage (CP) was highest in IV group. Out of 6 animals in each treatment group, a total of 3 successful deliveries were recorded in IV group (CP: 50%) (Fig. S1), followed by IC group with 2 positive deliveries (CP: 33.3%) (Fig. S2). The ABT group showed the lowest delivery rate with successful delivery of only one animal (CP: 16.6%) (Fig. S3).

## Discussion

The present study was conducted to examine the effectivity and optimum administration route of characterized allogenic AD-MSCs in promoting alleviation of chronic endometritis in buffaloes. As no previous studies were conducted for treatment of endometritis by AD-MSCs and that too in buffaloes, the decision on dosage of cells to be administered in the present study was established on results of the previous studies in equine and bovine with other diseases^34,38–40^. Allogenic AD-MSCs provide marvelous prospect of off-the shelf stem cell therapy especially for large herd of diseased animals where time is a limiting factor. The infused AD-MSCs translocate to the damaged tissues utilizing systemic as well as non-systemic homing mechanisms^41,42^, where they exhibit plethora of mechanisms to suppress pathogens, reduce inflammation and regenerate the damaged tissues. The present study, thus, analyzed both the intravenous and local routes to AD-MSC administration, in order to assess their comparative efficacies in treating endometritis. The health status of animals in abattoirs is often suspicious, therefore we derived the adipose tissues, for MSC isolation, surgically from healthy animals under observation. Several reports ^43–46^ demonstrated the rejection and adverse immunological reactions upon allogenic MSCs administration in animal models, making it absolutely necessary to evaluate the safety of allogenic administration of AD-MSCs before treatment of diseased animals.

Stem cells from adipose tissue are generally isolated by enzymatic method using collagenase. We developed a method of derivation of MSCs without the use of enzyme in order to avoid the enzymatic damage to the cells and reducing the cost of production. Our cell culture procedure involving explant culture yielded a homogenous population of AD-MSCs with healthy morphology (Fig. 1C). The isolated AD-MSCs exhibited all the mesenchymal stem cell markers as confirmed by cytochemical staining (ALP staining) (Fig. 2B), RT-PCR (Fig. 2M) and immunocytochemistry (Fig. 2D–L). Moreover, these cells were capable of multiple mesodermal lineage differentiation when conditioned with specific reagents and culture milieu (Fig. 2N–P). The cultured AD-MSCs were characterized by every possible parameter, in order to use them safely for the downstream administrations in animals.

In the present study, the safety analysis revealed fairly stable clinical and hematological parameters (Table 1) in healthy buffaloes upon administration of two consecutive doses of AD-MSCs separated by 7 days. Additionally, the basophils and ESR levels showed no significant variation during the experimental period of 15 days, which indicates the absence of any sign of clinical distress, substantial inflammation or adverse systemic immune reaction in animals after allogenic treatment by AD-MSCs. Our results are in line with the previous studies ^47–49^ which advocates the safety of allogenic MSC therapy even with repeated cell infusions. Nevertheless, the safety concerns, regarding the use of MSCs in allogenic therapies, raised by other studies ^43–46^ should not be overlooked and safety trials must be conducted prior to their allogenic usage.

During the efficacy analysis, the administration of AD-MSCs did not elicit any clinical distress as indicated by the relatively stable clinical variables between the days, in all the treatment groups (Table 2). CRP levels in the blood serum were found to decline significantly in IV and IC treatment groups after 15 days (Fig. 3C). However, higher percentage of animals with CRP values below 5% were recorded in IV group (data not shown) than IC group. It is well-established that CRP plays important functional role in inflammatory process and is, thus, a key acute systemic marker of underlying inflammation ^50^. The non-significance in CRP levels in ABT group of animals represents failure in inflammation reduction during the trial period. CRP is released by hepatocytes in response to increased levels of proinflammatory cytokine, *IL6* ^51^. Therefore, it is not surprising to expect a high CRP in serum in response to an increased *IL6* expression (Fig. 4C) in antibiotic (ABT) group, contrast to IV and IC groups. Similar results were obtained in vaginal discharge cytology, where IV as well as IC treatments significantly reduced the PMN counts, with no variation after ABT treatment. As PMNs are the first recruits of the acute inflammation ^52^, their population in the vaginal secretions inversely corresponds to the healing status of the tissue. Additionally, we observed a significant decline in VDS score in vaginal mucus of the IV and IC group buffaloes at day 15 in comparison with ABT at same time. For examining animals with clinical endometritis, a basic scoring method (Fig. 3A) based on the consistency of the vaginal mucus is widely employed to assess the severity of disease and is a potential prognostic marker for the effect of treatment ^9^. The decline in VDS from day 0 to day 15 corresponds to the reduction in inflammation in the IV and IC treatment groups. The animals in IV group delivered highest proportion of calves (CP: 50%) as compared to the IC (CP: 33.3%) and ABT (CP: 16.6%) groups. Despite the small sample size for the pregnancy data, a 50% calving percentage (in IV) may be viewed as a significant improvement, considering the previously recorded 50% decrease in conception rates due to endometritis ^53^ and a 35% success rate of artificial insemination in buffalo^54^. During the designing of the study, we have taken caution to opt the animals which have the past record of persistent endometritis (data shown in supplementary table) and multiple failed conceptions. At our farm, it has been observed that the animals having clinical endometritis are completely sterile and failed to deliver live births. Although there are scarce studies on buffalo endometritis, another group reported that the untreated endometritic buffaloes failed to conceive and deliver calf, which is in line with our observation^55^. Therefore, the sample size, although small, was a representative group of the endometritis affected buffaloes and improvement in fertility appears to be a promising outcome. The present study was originally a hypothesis-generating study and the data obtained will be used to design larger confirmatory studies.

Furthermore, absolute risk reduction refers to the actual difference in risk between the different interventions^56^, which was observed as highest (50%) in IV group, clearly demonstrating the healing efficacy of the intravenous treatment as compared to antibiotic treatment. According to the institutional ethics committee's guidelines, which forbid leaving ruminant animals untreated, the current study lacks a non-treatment control group. This leaves a possibility of self-healing in the animals irrespective of the treatment. However, achievement of 50% absolute risk reduction as well as a higher proportion of successful calf deliveries by subjects following artificial inseminations (Fig. S1), strongly justifies the healing potential of AD-MSCs administration through IV route. Surprisingly, we and the other research groups^34,57,58^ observed the positive effects of administering the small quantity of cells in the animals. Although reasons unknown, this observation may prove beneficial in rendering stem cell therapy a cost-efficient intervention. The antibiotic therapy was performed on cows following the standard protocols of the institutional farm, however, the sub-optimal response to antibiotic therapy points towards the possible antibiotic resistance among the animals selected under the study. This observation opens a rather alarming area for further investigational studies. Whatsoever may be the reason, the cows in MSC and EV groups recovered swiftly from the infected state.

Overall results of the study indicate towards the better healing and reduced inflammation in the animals administered with IV injections of AD-MSCs followed by IC administration. The expression of anti-inflammatory cytokines and antimicrobial peptides in the blood isolated during the course of different therapeutic interventions strongly advocates the effectiveness of IV administration. PI3, LCN2, CST3 and CATHL4 are the potent anti-microbial proteins produced by diverse range of innate immunity cells. The relatively high and stable expression of these genes in IV administered animals (Fig. 4A) throughout the treatment period, in contrast to other groups, demonstrates the efficacy of the treatment in decreasing the pathogen load. Although, MSCs have been demonstrated to inhibit the microbial growth via secretion of LCN2, CST3 and CATHL4^34^ the present results hint towards a possible mechanism where MSCs induce leucocytes to secrete these antimicrobial peptides. Leucocytes have been long known to secrete LCN2^34^ CST3^34^ and CATHL4^34^ in response to infections. MSCs have been found to regulate the production of inflammatory cytokines by inflammatory cells as well as activate immune cells to engage in phagocytosis and bacterial killing^59^. Similar to the parent cells, MSC derived vesicles have also been suggested to exhibit stimulation of immune system cells^60^. The CRP as well as PMN count presents the IC intervention almost equally effective as the IV treatment, but the expression of anti-microbial peptides in IC group is comparable to that of ABT group. Due to the lack of global gene expression analysis and small experimental group, the present study is inept to explain this anomaly in expression in IC group. One of the most important anti-inflammatory cytokines, produced via MSC-antigen presenting cell contact is IL10^61^, which was highly upregulated in IV group of present study. This common observation among other studies^62–64^ brands IL10, a key intermediate in healing by AD-MSCs. IL6 is a pro-inflammatory cytokine that causes increased systemic CRP levels. In concurrence with other studies^65,66^, its expression was significantly reduced after IV and IC AD-MSC injections (Fig. 4A,B). IL4 is a pleotropic cytokine whose expression by T-helper cells increase after interaction with MSCs^67^, functions mainly by suppressing the pro-inflammatory milieu. The significantly high and stable *IL4* expression after IV intervention in our study labels the intravenous route as a potent mode of AD-MSC administration. Consequently, we suggest to use the IV and IC routes of AD-MSC administration in concert, in order to mutually adjunct the positive effects of both the interventions. The underlying mechanisms of action of allogenic AD-MSCs on uterine physiology are still largely unknown and needs to be comprehensively explored in order to further increase their safety and efficacy.

## Conclusions

To the best of our knowledge, this is the first study involving the safety and efficacy of allogeneic treatment of AD-MSC in chronic endometritis affected livestock species. In the present study, administration of repeated doses of allogenic AD-MSCs to buffaloes did not elicit any immunological rejection or inflammation as well as nor did the clinical complications. The allogenic use of MSCs in buffaloes was found to be safe. Although, not considered in the present study, the species-specific as well as disease-specific optimal dose of AD-MSC administration should be standardized prior for more effective healing of animals. Moreover, employment of allogenic AD-MSCs in buffaloes though IV route was found to be most effective against suppression of endometritis in buffaloes. The observations of the study were limited by the small population of experimental animal and lack of global gene expression analysis. The present study is an endeavor towards transforming stem cell therapy into a routine commercial veterinary practice.

## Methods

### Chemicals and consumables

All the chemicals used in this study were purchased from Millipore Sigma, USA and cell culture plasticware/consumables were purchased from Thermo Scientific, USA unless otherwise stated.

### Surgical tissue sample collection

An intramuscular injection of local anesthetic, Lignocaine Hydrochloride (2% w/v), was administered as per the prescribed dosage in the loin region of the 27 months old buffalo calves (N = 6) from a local livestock shelter. Topical disinfectant (povidone-iodine) was applied on the site of surgery after shaving scrubbing followed by multiple sprays of 70% ethanol. An incision of around 3 cm was made in the skin and adipose tissue was sampled out and collected in DPBS containing 100 IU/mL penicillin, 100 mg/mL streptomycin and 2.5 mg/mL amphotericin B. The tissue samples were collected from 6 healthy animals, pooled together and were immediately transported to laboratory for further experiments. Using the lock-stitch pattern, suturing of incision was performed employing catgut and silk thread followed by application of antibiotic powder (Tetracycline Hydrochloride) on the surgical site. Post-surgery antibiotic injections (Ceftriaxone, 25 mg/kg body weight) were given for 3 days and antibiotic powder (Povidone Iodine) was applied for 5 days along with dressing of the area. Silk suture threads were removed after 10 days.

### Isolation, culture and characterization of adipose derived mesenchymal stem cells (AD-MSCs)

Adipose tissue was rinsed thrice in DPBS containing antibiotics (100 mg/L penicillin and 100 mg/L streptomycin) and minced into fine pieces (~ 2–3 mm). The dissected fat without any vascularization was collected and care was taken not to dissect out any fibrous tissue along with adipose tissue. The small pieces were embedded on the surface of tissue culture petri-dishes by applying pressure with forceps and cultured in DMEM/F-12 containing 10% FBS and 50 µg/ml gentamycin (called standard medium) at 38 °C, 5% CO_2_, and 95% relative humidity. After 2 weeks, the tissue segments were removed from the dishes and cells were cultured one additional week to reach confluence. Upon 80% confluence, cells were trypsinized (using 0.25% Trypsin-EDTA) and reseeded in T-25 flasks at a density of 5 × 10^4^/cm^2^ and incubated again at 38 °C, 5% CO_2_ and 95% relative humidity. The cells were also seeded in a separate 60 mm culture dish at density of 5 × 10^3^ cells/dish and cultured in pre-mentioned conditions for 2 weeks, in order to assess the colony forming potential of the cells. The cells at 3^rd^ passage were used to analyze the expression of mesenchymal stem cell specific markers by RT-PCR and immunostaining. The cells were also subjected to alkaline phosphatase (ALP) staining and in vitro differentiation into multiple mesodermal lineages (i.e. osteocytes, adipocytes and chondrocytes), which were confirmed by lineage specific biochemical stains. The detailed procedure of ALP staining, immunostaining, RT-PCR and in vitro differentiation assays are provided in supplementary file (methods: M1).

### Experimental animals and management

Ethical approval for the current study was obtained from Institute ethics committee. In compliance with the guidelines of the ethical committee, all experiments were carried out to ensure minimum possible suffering to animals. The study was performed on female buffaloes of Murrah breed (within 3–5 parity and 40–80 days of parturition) during March, 2020. All the animals were obtained from institutional farm and were allotted for the study by the institutional research committee. The animals which did not responded to the prior antibiotic therapies were opted for the efficacy study (supplementary table S3). Animals having other clinical diseases along with endometritis were not included in the study. The animals in different experimental groups were contained in separate loose housing system, which included a covered shelter with manger inside a paddock. All the animals had free access to clean drinking water. A standardized balanced feed consisting of dry fodder, green fodder and silage was supplied according to the animal’s physiological state.

### Endometrial cytology assessment by PMN leucocyte count

Endometrial samples (cervical discharge) were collected using a device created by modifying disposable cytobrush (Axcata-cybrush, New Delhi, India) for use in buffaloes^68^. After collection of samples from the genital tract, cytobrush was rolled on glass slides to make a uniform thin smear and air dried. The endometrial smears were fixed in methanol for 2–3 min, stained with field stain B (eosin 0.4%; lobachemie, India) for 15–20 s, rinsed with distilled water and stained with field stain A (methylene blue 0.5%; lobachemie, India) for 10 s. Slides were finally rinsed with water, air- dried and examined under the microscope (IX51, Olympus, Japan). At least 100 nucleated cells were counted in each slide and percentage of PMN leucocytes was calculated. The reference values established for cows^53,69^ were used as the cut-off values for PMN leucocyte to classify normal and endometritis affected animals, because the same for buffaloes are not established till date. Animals with cytobrush cytology showing ≤ 5% PMN leucocyte and clear cervical discharge were screened as normal. However, those with > 14% PMN leucocyte and turbid cervical discharge were marked as clinical endometritic.

### Administration of AD-MSCs for safety evaluation

For evaluation of treatment safety, 2 × 10^7^ cells/injection (AD-MSCs in 2 ml PBS) were introduced into the jugular vein (IV) and cervix (IC) of healthy buffaloes (N = 6) on day-0 and day-7. Post MSC administration, blood samples were collected from jugular vein at day-0, -1, -3, -7 and -15 for examination of clinical and hematological parameters. At day-0, blood samples were collected before the MSC administration. Clinical parameters included in the study were RR (respiratory rate), HR (heart rate) and RT (rectal temperature). The hematological parameters like hemoglobin, PCV (packed cell volume), leukocyte, erythrocyte, neutrophil, lymphocytes, monocytes and eosinophil levels were evaluated in order to examine any adverse effect of allogenic AD-MSCs treatment on buffaloes. The above-mentioned parameters were analyzed by a commercial diagnostic lab (PCC&VDL, Karnal, India).

### Treatment of endometritic animals using antibiotics and AD-MSCs for efficacy evaluation

For the efficacy analysis, animals with clinical endometritis, based on poly morphonuclear (PMN) leucocyte percentage (> 5%) and turbid vaginal discharge, were selected for experiments. Three different groups of diseased animals, marked as antibiotic (ABT), intravenous (IV) and intracervical (IC), were sorted comprising 6 animals in each group. On Day-0, cervical discharge and blood sample from jugular vein of the endometritis affected animals of all the experimental groups were collected. Thereafter, characterized AD-MSCs (at 3rd passage) were administered to groups IC and IV through injections containing 2 × 10^7^ cells in 2 ml PBS. Animals in group ABT were subjected only to intra-uterine infusion of Oxytetracycline (UltrOx, Neospark Drugs and Chemicals Pvt. Ltd., India) according to standard treatment procedure (3 gm/animal for 3 days) of the institutional farm^70^. The dosage of MSCs was repeated on day-7 in IV and IC groups. Evaluation of clinical parameters viz. RR, HR and RT was performed daily from day 1 to day 15. Additionally, blood samples were collected on day-0, -1, -3, -7 and -15 from jugular vein of all the animals for isolation of peripheral blood mononuclear cells (PBMC). The evaluation of C-reactive protein (CRP) and vaginal discharge score (VDS) was conducted on day-0 as well as day-15 serum and vaginal discharge samples respectively. CRP levels in serum were quantified by latex immune turbidity assay in a commercial diagnostic laboratory (PCC&VDL, Karnal, India). For evaluation of VDS, a score (on a scale of 0–3) was assigned to each vaginal fluid discharge based on visual turbidity and distinct characters (Fig. 3A). Further, absolute risk reduction (ARR) in endometritis was calculated by assuming VDS value of 0 as clinically cured (baseline) with 95% confidence interval (CI).

### Isolation of PBMC from blood samples

Lymphocytes were isolated from blood collected in EDTA coated vacutainers using Histopaque-1077. Three ml of blood was layered carefully upon 3 ml of histopaque in a centrifuge tube and centrifuged at 400 g for 30 min. The opaque ring at the plasma-histopaque interphase comprising lymphocytes and other mononuclear cells was aspirated carefully into another tube and 10 ml DPBS was added with gentle mixing. The tube was centrifuged again at 250 g for 10 min. Supernatant was discarded and cell pellet was resuspended in 5 ml of DPBS. Centrifugation with DPBS was repeated two times. The resulting pellet was stored in Trizol at − 80 °C for RNA extraction.

### Total RNA isolation, RT-PCR and quantitative RT-PCR

Total RNA was isolated from cultured MSCs and PBMCs using the TRIzol method. RNA quantification and quality check were performed using spectrophotometer (Infinite 200 NanoQuant, Tecan group Ltd., Switzerland). First-strand cDNA was synthesized from 500 ng RNA using RevertAid First Strand cDNA Synthesis Kit (Invitrogen, USA), according to the manufacturer's manual. All the gene specific primer pairs (Table S1) were designed manually (using OligoCalc) from two different exons of the gene, employing BLAT (BLAST-like alignment tool, UCSC). Conserved sequences from different transcript variants (employing ClustalW) were utilized for primer designing from the *Bubalus bubalis* CDS sequences available in KEGG database. The PCR was performed on thermocycler (S1000TM Thermal Cycler, BioRad, USA) using Dream Taq Green PCR master mix (Invitrogen, USA) according to manufacturer’s instructions. The PCR reaction conditions were, initial denaturation at 95 °C for 5 min, 35 cycles of denaturation at 95 °C for 20 s followed by annealing at 61 °C for 20 s and extension at 72 °C for 20 s. Subsequently, a final extension at 72 °C for 5 min. The amplified products were loaded onto agarose gel, electrophoresed, and photographed on gel imaging system (Gel Doc TM XR+, Bio-Rad, USA). The amplified products were loaded onto agarose gel, electrophoresed, and photographed on gel imaging system (Gel Doc TM XR+, Bio- Rad, USA). Quantitative PCR was performed on LC-480 light cycler (Roche, Germany) using DyNAmo SYBR green (Thermo Fisher Scientific, USA). The qPCR program consisted of 40 cycles of denaturation at 95 °C for 10 s, annealing at 61 °C for 15 s, and extension at 72 °C for 20 s. Glyceraldehyde 3-phosphate dehydrogenase (*GAPDH*) was used as normalizing control. Each experiment was repeated independently at least three times, and the fold change in the expression of each gene was analyzed employing the 2^−ΔΔCt^ method.

### Artificial insemination of animals

After the end of 15-day experimental period, the animals pertaining to all the treatment groups were subjected to artificial insemination. The animals were estrus synchronized by Prostaglandin F2α administration and inseminated according to am-pm rule using frozen-thawed bull semen at institutional farm. The animals were followed for entire pregnancy period until successful delivery of calves.

### Statistical analysis

The clinical and hematological data in safety and efficacy analysis were analyzed using repeated measures one-way analysis of variance (ANOVA). The VDS, PMN count and CRP levels values were analyzed by two-tailed paired t-test. The qPCR data was analyzed using one-way ANOVA with Dunnett post hoc test. The entire statistical analysis was performed using GraphPad PRISM 8 (GraphPad Software Inc, CA, USA). All experiments were repeated at least three times. Variations between replicates are indicated with the standard error mean (± SEM in error bars on graphs). The statistical significance was set at *p* ≤ 0.05, 0.01, and 0.001.

### Ethics approval

The study was conducted according to the ARRIVE guidelines of pre-clinical trials and approved by the Institutional Review Board (or Ethics Committee) of National Dairy Research Institute, Karnal, India with approval no. 42-IAEC-18-24, dated-30th June 18.

## Supplementary Information

Supplementary Information.

## Data Availability

The datasets generated during and/or analyzed during the current study are available from the corresponding author on reasonable request.
