# Supplementary material for: Allogenic adipose derived mesenchymal stem cells are effective than antibiotics in treating endometritis
Source: Sci Rep. 2023 Jul 12;13:11280. doi: 10.1038/s41598-023-36820-y (PMC10338491; doi:10.1038/s41598-023-36820-y)
Supplement: Supplementary file 1 — Supplementary Information. [file 41598_2023_36820_MOESM1_ESM.pdf]

Supplementary File

Allogenic adipose derived mesenchymal stem cells are effective than antibiotics in treating endometritis

Vinay Bhaskar[1], Sikander Saini[1], Shama Ansari[1], Shubham Ghai[1], Abhishek Thakur[1], Suman Chopra[1], Vivekananda Verma[1], Dhruba Malakar[1]*

[1] National Dairy Research Institute, Karnal, India-132001

* Correspondence: dhrubamalakar@gmail.com; Tel.: +91-9416741839

Cell and Molecular Biology Lab, Animal Biotechnology Centre

National Dairy Research Institute, Karnal, India-132001

Supplementary File

Supplementary File Information

M1: Characterization of AD-MSCs. Figure S1: The buffaloes from IV group that calved after artificial insemination; Figure S2: The buffaloes from IC group that calved after artificial insemination; Figure S3: The buffalo from ABT group that calved after artificial insemination; Table S1: Details of primers used in the study; Table S2: Cervical vaginal discharge score allocated to each animal in IV, IC and ABT, on day 0 (D0) and day 15 (D15); Table S3: History of antibiotic treatments in animals before opting for the study.

I. Methods

M1: Characterization of AD-MSCs.

1. Immunostaining of AD-MSCs

MSCs were cultured in 4-well dish and fixed with 4% paraformaldehyde in DPBS for 30 min and washed 3 times with DPBS. Cells were incubated with the 4% Bovine Serum Albumin (blocking solution) for 30 min at RT followed by an overnight incubation with primary antibodies (1:100) (Table S1) against CD105, CD90, and CD73 at 4 °C. After repeated washings with DPBS cells were incubated with FITC-labelled secondary antibodies (Thermo Scientific, USA) at 1:500 dilution for 1 hr at RT. Thereafter, cells were co-stained with nuclear dye, Hoechst-33342 in DPBS for nuclei visualization. Cells were finally washed 3 times in DPBS and examined under the microscope (IX51, Olympus, Japan) equipped with epifluorescence.

2. Alkaline phosphatase (ALP) staining

AD-MSCs cultured for 3rd passage were used for analysis of ALP activity by Alkaline Phosphatase Detection Kit as per manufacturer's instruction manual. Briefly, the culture medium was aspirated, cells were washed with DPBS, and fixed with 4% Paraformaldehyde in DPBS for 1-2 minutes. Thereafter, fixative was aspirated out and cells were washed with

DPBS. A mixture containing Fast Red Violet: Naphthol AS-BI phosphate solution: water (2:1:1 ratio) was added to the cells and incubated in dark at RT for 15 min. The staining solution was aspirated and cells were rinsed with DPBS. The stained cells covered with DPBS were photographed under inverted microscope (IX51, Olympus, Japan).

3. Osteogenic differentiation of AD-MSCs and alizarin red staining

After the 3rd passage, AD-MSCs were seeded in multi-well plate at a density of 15,000 cells/cm^2 and incubated for 48-72 hrs to attain confluence, following replacement of standard medium with differentiation medium. The osteogenic medium was comprised of standard medium supplemented with 10% FBS, 100 nM dexamethasone, 200 mM ascorbic acid and 10 mM of b-glycerophosphate. After replacement with osteogenic differentiation medium, cells were incubated for 21 days at 38 °C, 5% CO_2, and 95% relative humidity and the medium was replaced after every 72 hrs. Finally, cells were washed in DPBS, fixed in 95% methanol for 10 min, stained with 2% alizarin red S solution for 5 min, rinsed again with water and examined for Ca^{2+} deposits using inverted microscope (IX51, Olympus, Japan).

4. Adipogenic differentiation of AD-MSCs and oil red O staining

The cells were cultured on fibronectin-coated multi-well plates until confluence as mentioned previously for osteogenic differentiation and standard culture medium was replaced by adipogenic differentiation medium prepared by supplementing 100 nM dexamethasone, 80 mM indomethacin, 0.5 mM 3-isobutyl-1-methylxantine, and 10 mg/ml insulin in standard culture medium. The cells were incubated with the differentiation medium at 38 °C, 5% CO_2, and 95% relative humidity for 21 days with regular replacement of medium as described earlier. At the end of incubation period, cells were fixed with 4 % paraformaldehyde, washed with DPBS, and stained with oil red O. Finally, the cells were visualized under inverted microscope (IX51, Olympus, Japan).

5. Chondrogenic differentiation of AD-MSCs and toluidine staining

Supplementary File

As described above, the standard culture medium in MSCs at the 3rd passage was replaced with high-glucose DMEM supplemented with 10% FBS, 0.05 g/l ascorbic acid, 100 nM dexamethasone, 1% ITS, and 3.7 g/l sodium bicarbonate and incubated at standard culture conditions (38 °C, 5% CO_2, and 95% relative humidity) for 21 days with regular medium replacement at 72 hrs. After 21 days chondrogenic differentiation cells were washed with DPBS and fixed using 4% paraformaldehyde for 15 min and rinsed twice with DPBS. The cells were, then, stained with toluidine blue dye for 6-7 min at room temperature and rinsed twice with DPBS. Toluidine blue dye is specific for glycosaminoglycans and proteoglycans. Finally, the cells were photographed under inverted microscope (IX51, Olympus, Japan).

Supplementary File

II. Figures

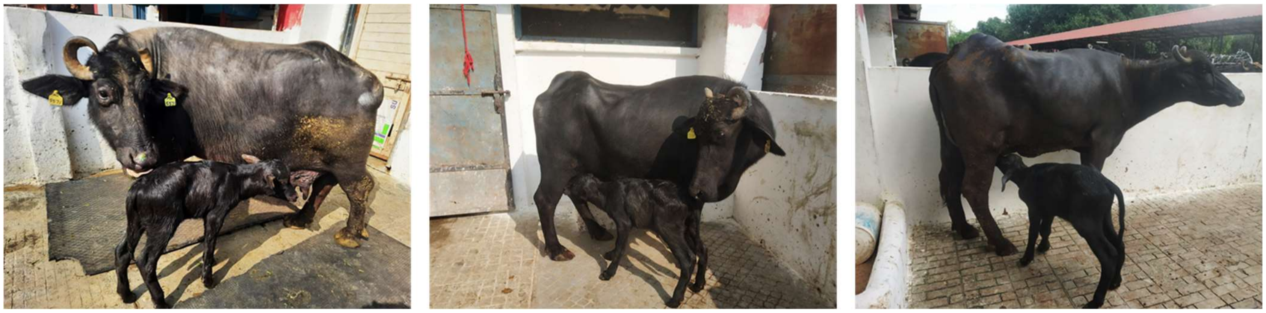

Figure S1: The buffaloes from IV group that calved after artificial insemination.

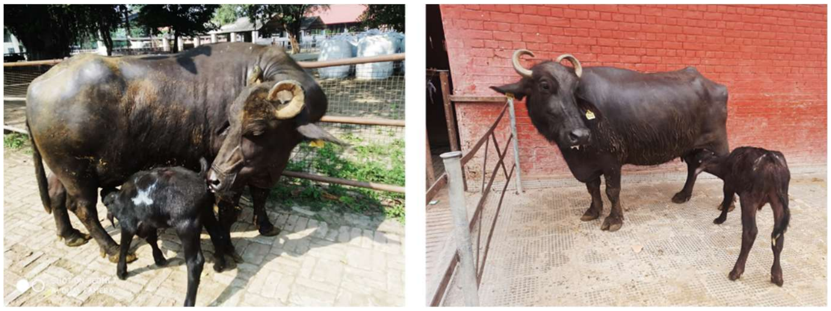

Figure S2: The buffaloes from IC group that calved after artificial insemination.

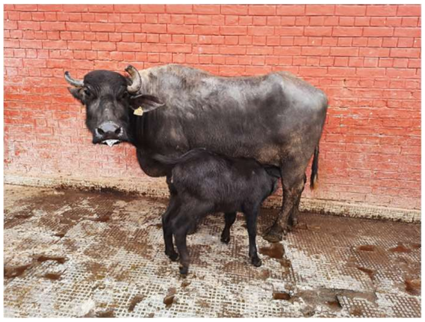

Figure S3: The buffalo from ABT group that calved after artificial insemination.

Supplementary File

III. Tables

Table S1: Details of primers used in the study.

S.No.	Name of the Gene	Primers	Sequence Primers	Product length (bp)
1	*CD73*	Fw	CACACCAACGACGTACACAGCCG	202
		Rv	TCGGTGCCCTTGTACACGGTGAAC	
2	*CD90*	Fw	CAGTATGAGTTCAGCCTGACCCGTGA	221
		Rv	CACAGAGACATCCTTATTGGAGACGGAG	
3	*CD105*	Fw	GTGCTCCAGCGATGGCATGACTCT	200
		Rv	AGTCTGATGACCACCTCGTTACTGACC	
4	*CD34*	Fw	CTCAGGACAGCAATGGGACCACAG	195
		Rv	AAAGTGCTGGGCTGCAGAGTCGC	
5	*CD45*	Fw	GGTGACTTCTGGCAGATGGTCTTCC	197
		Rv	GGAGTGTCTCAGTTCAAATGCACGGAC	
6	*CD79a*	Fw	GGAGGGCACCAAGAACAACATCATCAC	174
		Rv	GTCATCAAGGTTCAGGCCCTCATAGAGA	
7	*GAPDH*	Fw	GTCTTCACTACCATGGAGAAGG	139
		Rv	TCATGGATGACCTTGGCCAG	
8	*CATHL4*	Fw	CTACTGCTGCTGGGACTAGTGGTG	252
		Rv	CCATTCTCCTTGAAGTCACACTGCTCTG	
9	*IL10*	Fw	CTGTTGACCCAGTCTCTGCTGGATG	231
		Rv	CCTTCTCCACCGCCTTGCTCTTGT	
10	*IL6*	Fw	GACCTGCTGGAGAAGATGCAGTCTTCAA	185
		Rv	CCAGTGGACAGGTTTCTGACCAGAG	
11	*IL4*	Fw	CTGCCCCAAAGAACACAACTGAGAAGG	128
		Rv	CAAGCT TT AG TTCCTGTCGAGTCC	
12	*CST3*	Fw	CCTGATGGAGGCGGACGTCAATGA	242
		Rv	CTCTTCAGGTGCGGCTGGTTATGGAA	
13	*LCN2*	Fw	CTCCAACTACGAGCTGAAGGAAGACG	198
		Rv	GGCAAACTGGTTGTAGTCGGTGTTCAC	

Table S2: Cervical vaginal discharge score allocated to each animal in IV, IC, and ABT on day 0 (D0) and day 15 (D15).

IV			IC			ABT		
Buffalo number	D0	D15	Buffalo number	D0	D15	Buffalo number	D0	D15
7168	1	0	7046	1	0	6556	3	3
5571	3	0	7043	2	1	5834	3	2

Supplementary File

7378	3	1	6675	3	2	7481	2	2
7019	3	1	6109	3	1	6388	3	2
6050	1	0	7713	2	2	7131	2	2
6281	2	1	7755	3	1	7696	2	2

Table S3: History of antibiotic treatments in animals before opting for the study.

S.No.	Buffalo No.	Dates of antibiotic therapies prior to experiment
1	7168	15/12/2019, 07/1/2020, 19/1/2020, 29/1/2020 (Total 04 times)
2	5571	29/05/2019, 19/08/2019, 15/10/2019, 5/11/2019, 29/01/2020, 11/02/2020 (Total 06 times)
3	7378	14/10/2019, 8/12/2019, 10/12/2019, 19/01/2020 (Total 04 times)
4	7019	06/11/2019, 04/01/2020, 07/02/2020 (Total 03 times)
5	6050	14/08/2019, 29/10/2019, 4/01/2020, 20/01/2020, 7/02/2020 (Total 05 times)
6	6281	01/05/2019, 23/05/2019, 07/09/2019, 01/11/2019, 23/12/2019, 10/01/2020, 20/01/2020, 06/02/2020 (Total 08 times)
7	7046	07/12/2019, 31/12/2019, 23/01/2020 (Total 03 times)
8	7043	01/12/2019, 20/12/2019, 02/01/2020 (Total 03 times)
9	6675	15/11/19, 02/12/19, 24/12/2019, 29/01/2020 (Total 04 times)
10	6109	22/1/2020, 6/2/2020, 26/2/2020 (Total 03 times)
11	7713	03/08/2019, 23/11/2019, 03/12/2019, 02/01/2020, 01/02/2020 (Total 05 times)
12	7755	07/09/2019, 15/10/2019, 05/11/2019, 11/01/2020, 08/02/2020, 20/02/2020 (Total 06 times)
13	6556	24/07/2019, 14/08/2019, 15/10/2019, 21/12/2019, 25/01/2020 (Total 05 times)
14	5834	19/08/2019, 17/10/2019, 03/11/2019, 22/11/2019, 11/01/2020 (Total 05 times)
15	7481	14/08/2019, 01/11/2019, 11/12/2019, 13/01/2020 (Total 04 times)
16	6388	30/10/2019, 07/12/2019, 23/12/2019, 18/01/2020 (Total 04 times)
17	7131	24/12/2019, 27/01/2020, 28/02/2020 (Total 03 times)
18	7696	23/12/2019, 05/01/2020, 24/02/2020 (Total 04 times)
